# Recurrent Primary Hyperparathyroidism in Hyperparathyroid Jaw Tumor Syndrome: A Case Report

**DOI:** 10.7759/cureus.42732

**Published:** 2023-07-31

**Authors:** James R Kang, Jacob Burlew, Andrew Paulus, Joseph Kluesner

**Affiliations:** 1 Internal Medicine, Wright State University Boonshoft School of Medicine, Dayton, USA; 2 Internal Medicine, Wright-Patterson Air Force Base, Dayton, USA; 3 Endocrinology, Wright-Patterson Air Force Base, Dayton, USA

**Keywords:** familial hyperparathyroidism, adult primary hyperparathyroidism, cdc73 gene, hyperparathyroidism-jaw tumor syndrome, hyperparathyroid

## Abstract

Hyperparathyroidism occasionally develops secondary to genetic disorders, though CDC73-related diseases such as hyperparathyroid jaw tumor syndrome (HPT-JT) are among the least common. Typically, patients are identified in the third decade of life by characteristically early development of hyperparathyroidism, atypical pathology on histologic evaluation of parathyroid adenomas, or presence of concomitant tumors in the jaw, renal, or uterine anatomy. We report a case of a 60-year-old male who was identified as having hyperparathyroidism jaw tumor syndrome after presenting with recurrent hyperparathyroidism 10 years after parathyroid adenoma resection, which is likely the oldest age to date for diagnosis. We also review his clinical presentation, pathophysiology, genetic significance, and surveillance criteria relating to HPT-JT.

## Introduction

Hyperparathyroidism (HPT) may develop through several different pathological mechanisms which are typically described as primary (overproduction of the parathyroid hormone, or PTH, at the level of the parathyroid glands), secondary (appropriately increased level of PTH in response to a stimulus), or tertiary (longstanding secondary HPT leading to excessive secretion of PTH). Primary hyperparathyroidism (PHPT) in North America has roughly equal incidence in men and women before 45 years of age and a higher incidence in women after 45 years of age. Secretion of PTH is normally regulated via a negative feedback loop in response to serum calcium levels. Normal physiologic response to high serum calcium levels is a down-regulation of PTH secretion in order to decrease PTH activity in the bones, thereby decreasing bone resorption, and in the kidneys, decreasing calcium reabsorption. Decreased PTH secretion also decreases 1alpha-hydroxylase production in the kidneys, leading to decreased active vitamin D (1,25-dihydroxycholecalciferol) and consequently reduced calcium absorption in the small intestines [1]. Provided there are no contraindications to surgery, parathyroidectomy can be considered in all PHPT patients who meet at least one of the following criteria regardless of the presence of symptoms: serum calcium >1 mg/dL above the upper limit of normal, skeletal involvement with vertebral fracture or decreased bone mineral density, renal involvement with decreased renal function (glomerular filtration rate (GFR) <60 mL/min), nephrocalcinosis/nephrolithiasis, hypercalciuria, or age <50 years [2]. It is also worth noting that most patients with primary HPT present with little to no symptoms. Hyperparathyroidism jaw tumor syndrome (HPT-JT) is a rare autosomal dominant genetic disorder seen in individuals with a mutation of the CDC73 gene. This disorder is not commonly discovered unless through genetic screening of affected patients and their families. Among those who are affected by HPT-JT, only about 30% of them develop jaw tumors [3]. Additionally, pathologic variants of CDC73 have been associated with tumors of the kidneys, uterus, and parathyroids, as well as renal cysts. The current clinical practice guidelines suggest genetic screening for patients who either develop HPT before 30, have multiglandular disease, or have family history of hypercalcemia and/or syndromic disease [2]. The following case report describes a rare instance of HPT-JT identified in an asymptomatic patient without tumors or known family history of CDC73 mutation at 60 years old.

## Case presentation

A 60-year-old male patient was found to have hypercalcemia and elevated PHPT on routine lab work 10 years status post parathyroid adenoma resection and was directed to follow up with endocrinology. He was otherwise clinically asymptomatic with elevated PTH. The patient’s family history was notable for PHPT in his brothers; he denied any family history of jaw, uterine, or renal tumors. Preoperative PTH levels of over 600 pg/mL (lab reference range 7.5-53.5 pg/mL) prompted genetic screenings for multiple endocrine neoplasia (MEN) and RET mutations, which were negative. Surgical pathology from the parathyroidectomy demonstrated increased mitotic count, moderately elevated proliferative index, a focus suspicious for perineural involvement, pockets of cells with macronucleoli, and intralesional fibrosis. Despite these concerning features as well as the degree of preoperative hypercalcemia, there was no evidence of metastasis or definitive vascular invasion, and therefore insufficient evidence to classify the lesion as parathyroid carcinoma. The final pathologic diagnosis was hypercellular parathyroid tissue with atypical features most consistent with an atypical parathyroid adenoma. Subsequent postoperative labs consistently demonstrated normalization of calcium and PTH levels.

**Figure 1:**
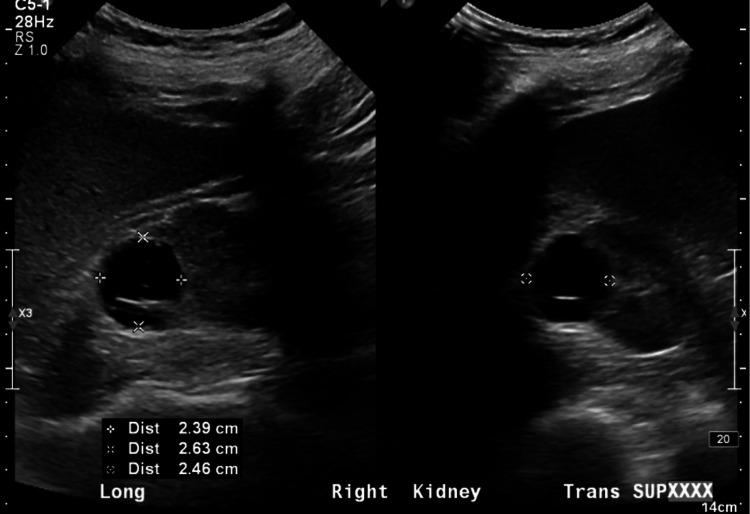
The patient was noted to have multiple renal cysts bilaterally. The largest cyst noted on a right kidney ultrasound measured 2.39 cm x 2.63 cm x 2.46 cm.

**Figure 2:**
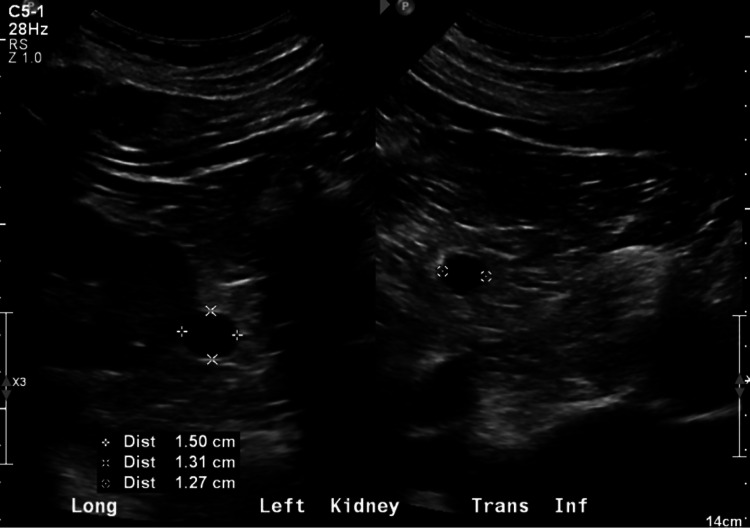
The patient was noted to have multiple renal cysts bilaterally. The largest cyst noted on a left kidney ultrasound measured 1.5 cm x 1.31 cm x 1.27 cm.

After a decade of stability, the patient’s PTH again became elevated to 87.5 pg/mL with a repeat level of 118.6 pg/mL, prompting endocrinology consultation (Table 1). Renal function panels including serum calcium levels were within normal limits on both occasions. He subsequently underwent investigation of secondary causes of HPT, including a serum vitamin D level of 78.5 ng/mL and a 24-hour urine calcium of 179 mg/24 hr. He was given a diagnosis of normocalcemic hyperparathyroidism. In the setting of his family history of PHPT as well as the prior pathology demonstrating an atypical adenoma, he was referred to genetics evaluation which demonstrated a pathologic mutation at CDC73 (c.376C>T(p.Arg126)). A subsequent neck ultrasound demonstrated no evidence of a parathyroid adenoma, while renal ultrasound demonstrated bilateral renal cysts (Figures 1, 2). Dual X-ray absorptiometry (DEXA) scan and spine plain films were unremarkable for osteoporosis or compression fracture. Given the above findings, the patient did not meet surgical criteria for his PHPT.

**Table 1:** The patient's laboratory workup performed 10 years following his parathyroid adenoma resection demonstrated elevated serum PTH levels with normal GFR, serum vitamin D, calcium, and 24-hour urine calcium levels. PTH: parathyroid hormone; GFR: glomerular filtration rate

Laboratory test	Value	Units	Reference range
Initial PTH 10 years post-op	87.5	pg/mL	7.5-53.5 pg/mL
Repeat PTH 10 years post-op	118.6	pg/mL	7.5-53.5 pg/mL
Initial serum calcium level 10 years post-op	9.4	mg/dL	8.4-10.2 mg/dL
Repeat serum calcium level 10 years post-op	8.9	mg/dL	8.4-10.2 mg/dL
Initial GFR 10 years post-op	77	mL/min	>60 mL/min
Repeat GFR 10 years post-op	77	mL/min	>60 mL/min
Serum vitamin D level 10 years post-op	78.5	ng/mL	30-100 ng/mL
24-hour urine calcium level 10 years post-op	179	mg/24 hr	100-300 mg/24 hr

## Discussion

We present a 60-year-old male whom we newly diagnosed with HPT-JT after a recurrence of hyperparathyroidism. This case is particularly unusual given our patient's age of diagnosis at 60 and may be the oldest age at diagnosis of HPT-JT to date, with the median age at diagnosis being 27 (mean of 32-36) years old [4]. Autosomal dominant CDC73-associated HPT-JT presents with less disruptive symptoms, and isolated PHPT with mutations predisposes patients to missed diagnosis. The patient’s recurrence of PHPT was also notable for normocalcemia, indicating future surveillance with electrolytes alone may be insufficient. In this case, the patient’s family history of primary hyperparathyroidism and surgical pathology with atypical features were the trigger for genetic screening. The patient was ultimately referred to dentistry for panoramic dental X-rays which were normal (to be repeated every five years), and to urology for further revaluation of his renal cysts. He was also encouraged to have his family members receive genetic testing and counseling. 

Since CDC73-associated disease typically does not manifest with significant symptoms, the incidence of this disease is likely under-reported. A review of recent medical literature suggests an estimated 200 reported cases of HPT-JT since the syndrome was first diagnosed in 1958 [5]. We identified 21 unique case reports of HPT-JT in the past 10 years via a PubMed search using the terms "HPT-JT" and "CDC73." The PubMed search resulted in a wide variety of described findings including renal cysts, mixed epithelial and stromal tumors, recurrent fibro-osseous jaw lesions, and parathyroid adenomas and carcinomas [6-9]. Forty-eight total patients were described in these reported cases. Among them, 14 female patients and 10 male patients were identified by both sex and age, with the average age at diagnosis being 33.4 years for female patients and 32.4 years for male patients. The youngest female patient reported was 19 years old and presented with mandibular ossifying fibromas, while the youngest male patient reported was six years old and presented with multiple maxillary and mandibular ossifying fibromas [10]. The oldest reported female patient in our search was 52 years old with adenomyosis and the oldest reported male patient was 57 years old with brown tumors secondary to abnormal metabolism of the bone from HPT [11,12]. One case report described 13 family members with a pathologic mutation of the CDC73 gene and found the penetrance to be roughly 50% at 40 years of age [13]. There did not appear to be a significant difference between male and female patients in the associated findings as related to their respective HPT-JT, with the obvious exception of abnormal female genitourinary pathologies such as adenomyosis and ovarian tumors.

The lower prominence of associated jaw tumors in HPT-JT cases contradicts the syndrome's nomenclature. Still, it is worth noting some distinguishing characteristics of these jaw tumors. Fibro-osseous jaw lesions associated with HPT-JT typically occur prior to the third decade of life and are benign, but they can cause significant functional limitations through destruction of local tissues and often prompt surgical removal. These particular lesions differ from most other sporadic causes of jaw fibromas by their recurrent nature, potential bilaterality, and radiolucency (as opposed to mixed radiolucency/radioopaque) on X-ray [6,8].

The penetrance of the CDC73 mutation is unknown and genetic counselors estimate each child of an affected individual has a 50% chance of inheriting a clinically meaningful variant [5]. Diligence regarding family history, atypical surgical pathology, and screening for patients with PHPT diagnosis before age 30 in line with current guidelines ensures optimal patient outcomes, and this case emphasizes that genetic screening should be considered even when patients are significantly older than the average age of HPT-JT diagnosis. Once patients have been identified, serial monitoring will be required subsequently via labs (annual serum calcium, intact parathyroid hormone, 25-(OH) vitamin D) and imaging every five years with dental panoramic X-rays and renal ultrasounds. For women, routine gynecologic examinations and screening suffice for surveillance on the standard schedule recommended by the US Preventive Services Task Force.

## Conclusions

HPT-JT is a rare genetic disorder that affects multiple organ systems and patients are at high risk for both malignancies and consequences of hypercalcemia. This case report emphasizes the significance of pursuing genetic testing in patients with guideline-recommended indications, such as in this case where a strong family history of HPT and atypical parathyroid pathology led to diagnosis of HPT-JT. Expeditious diagnosis can lead to appropriate treatment of patients and their affected family members to mitigate consequences of HPT as well as renal, jaw, and uterine tumors even at late age of diagnosis. This is especially important for older patients whose disease may be mistaken for uncomplicated PHPT. We recommend review of prior parathyroid surgical pathology as well as screening for familial history of endocrine disorders and/or specific tumors for all patients, as well as continued surveillance for urologic, dental, and other associated disorders for those with identified HPT-JT mutations to reduce morbidity from CDC73-related disease.
